# Responses of Lowland Rice Genotypes under Terminal Water Stress and Identification of Drought Tolerance to Stabilize Rice Productivity in Southern Thailand

**DOI:** 10.3390/plants10122565

**Published:** 2021-11-24

**Authors:** Tajamul Hussain, Nurda Hussain, Mukhtar Ahmed, Charassri Nualsri, Saowapa Duangpan

**Affiliations:** 1 Laboratory of Plant Breeding and Climate Resilient Agriculture, Agricultural Innovation and Management Division, Faculty of Natural Resources, Prince of Songkla University, Songkhla 90110, Thailand; 6110630006@psu.ac.th (T.H.); 6310120017@psu.ac.th (N.H.); 2 Energy Technology Program, Faculty of Engineering, Prince of Songkla University, Songkhla 90110, Thailand; 3 Department of Agronomy, Faculty of Crop and Food Sciences, PMAS Arid Agriculture University, Rawalpindi 46300, Punjab, Pakistan; ahmadmukhtar@uaar.edu.pk; 4 Agricultural Innovation and Management Division, Faculty of Natural Resources, Prince of Songkla University, Songkhla 90110, Thailand; ncharass@yahoo.com

**Keywords:** lowland rice, terminal water stress, grain yield, stress indices, stress tolerance

## Abstract

Lowland rice is an important cereal crop that plays a key role in the food security and the economy of Thailand. Terminal water stress (TWS) in rainfed lowland areas poses threats to rice productivity due to stress occurrence at terminal crop stages and extreme sensitivity of rice to TWS. A two-year study was conducted to characterize the performance of yield and yield attributes of twelve Thai lowland rice genotypes under TWS, to identify stress-tolerant genotypes using stress response indices and to identify promising stress indices which are correlated with grain yield (GY) under well-watered (WW) and TWS conditions for their use as rapid identifiers in a rice crop breeding program for enhancing drought stress tolerance. Measurements were recorded under WW and TWS conditions. Highly significant variations were observed amongst assessed genotypes for their yield productivity responses. According to stress response indices, genotypes were categorized into stress-tolerant and stress susceptible genotypes. Genotypes Hom Pathum, Sang Yod, Dum Ja and Pathum Thani-1 were found highly stress tolerant and relatively high yielding; genotypes Look Pla and Lep Nok were stress tolerant, whereas genotypes Chor Lung, Hom Nang Kaew and Hom Chan were moderately tolerant genotypes. Hence, stress-tolerant genotypes could be potentially used for cultivation under rainfed and water-limited conditions, where TWS is predicted particularly in southern Thailand to stabilize rice productivity. Stress tolerance indices, including stress tolerance index (STI), geometric mean productivity (GMP), mean productivity index (M_PRO_) and harmonic mean index (M_HAR_), indicated strong and positive associations with GY under WW and TWS; thus, these indices could be used to indicate stress tolerance in rice crop breeding program aimed at a rapid screening of lowland rice genotypes for stress tolerance.

## 1. Introduction

Rice is an important cereal after wheat that contributes to food security worldwide [1]. However, water stress has limited the production of both cereal crops [2]. Lowland rice systems contribute a major portion of rice production [3], and rainfed lowland rice is cultivated on approximately 6.2 million hectares worldwide [4]. In Thailand, rice is a major crop contributing to the food security and economy of the country. Even though rice production in southern Thailand contributes only 6% of the total rice production [5], it is of great importance to the regional food security. Rainfed lowland rice is a major production system in southern Thailand. However, rainfed lowland rice production systems are extremely vulnerable and variable in nature as water stress can occur at any crop growth stages. Climate change has also caused an increase in temperature and fluctuations in rainfall occurrence leading to regular heat and drought stress intervals [6,7]. Water stress is considered an important abiotic stress deleteriously affecting field crop productivity [6,8]. Rainfed lowland rice is cultivated in the rainy season in Thailand [7,9]. Due to seasonal variations in rainfall and occurrence of WS at different crop developmental stages, lowland rice production is drastically affected.

Water stress occurrence is critical under rainfed conditions as it affects plant growth and development [10]. Occurrence of water stress at various crop growth stages negatively influences the performance of specific attributes [11], leading to declined yield [12]. Timing of stress occurrence during early growth, mid-season and at terminal stages impact on severity of yield losses [13]. A stress event at early rice growth stages has an influence on leaf numbers and size, tillering capacity and stem height and affects panicle development, ultimately resulting in a reduced yield [14,15]. Water availability after the stress interval at the early growth stage helps plants recover, leading to lesser loss in yield. However, terminal water stress (TWS) intervals highly influence plant performance and lessens the chances of recovery to occur, leading to increased yield losses as rice is extremely sensitive to TWS [16]. TWS delays various plant development stages including panicle initiation and flowering [17], leading to spikelet sterility and reduction in number of panicles [18]. In addition, TWS causes abortion of ovules, deteriorates the grain filling process and alters source to sink distribution of assimilates, leading to reduced grain yield (GY) [19,20]. 

Stress-tolerant genotypes are genotypes that have the potential to maintain higher productivity under water stress [21]. Due to the extreme sensitivity of rice to TWS, different rice genotypes exhibit differential responses [10,18,22]. In the perspective of farmers, a stress-tolerant genotype is that which is highly capable of maintaining yield under limited water availability [23]. Therefore, high yielding genotypes under a diverse range of environments are desired and the cultivation of such genotypes could help to maintain rice productivity [2]. The GY of stress-tolerant genotypes is less affected under water stress as compared to stress susceptible genotypes. Cha-um et al. [24] reported that panicle size and filled grains of two stress tolerant rice genotypes were not significantly reduced as compared to two stress susceptible genotypes. According to Ichsan et al. [2], there are various local genotypes used by farmers around the world that have tolerance against water stress, in addition to stress-tolerant genotypes developed by research institutions and organizations. To enhance the resistance of rice against water stress, these genotypes are potential sources of germplasm, which are available in each growing season. In addition, it was observed that wild genotypes exhibited less decline and maintained GY under water stress as compared to cultivated genotypes [25]. Therefore, the identification and cultivation of stress tolerant genotypes from local germplasm could help to stabilize productivity under terminal water stressed environments. 

Several techniques and procedures are used to study water stress tolerance in rice genotypes at different crop growth stages [14,18,26,27]. A drought stress scoring method was used as the main criteria for the assessment and selection of rice cultivars for stress tolerance at reproductive crop growth stages in field trials [28] and genotypes producing high yields under water stress were selected as stress-tolerant genotypes. Numerous stress tolerance indices have been used [6,29,30,31,32,33,34,35,36,37,38] based on mathematical association among yield production under well-watered (WW) and water stressed conditions. According to Clarke et al. [38] and Fernandez [32], stress indices are generally based on the stress sensitivity or stress tolerance of tested genotypes. In the selection of stress tolerant genotypes, these indices provide the effect of water stress based on yield losses occurring under stress as compared to optimal or WW conditions [39]. The relative yield performance of a specific genotype in comparison to other tested genotypes under the same water stress indicates stress tolerance [40], and measure of reduction in yield under stress refers to the stress susceptibility of a genotype [41]. The stress susceptibility index (SSI) for a genotype was suggested by Fischer and Maurer [37], whereas geometric mean productivity (GMP) and stress tolerance index (STI) were proposed by Fernandez [32]. The mean productivity (M_PRO_) index is an average yield under WW and water stressed conditions [33]. Harmonic mean index (M_HAR_) was suggested by Schneider et al. [34]. The tolerance index (TI) is the difference in productivity between WW and water stressed conditions [35]. The yield stability index (YSI) was defined by Bouslama and Schapaugh [36]. All these indices have been used widely and are proposed in drought stress tolerance studies. However, the positive or negative associations of these indices with GY may vary. The significant differences among various indices were reported by Golabadi et al. [42] and Saba et al. [43] except SSI. Significant positive associations for GY under WW and stress indices (GMP, MP, STI, YSI, TOL and YI) and GY under water stressed conditions and stress indices (STI, GMP, MP, YSI and YI) have been observed by Golabadi et al. [42] and Arif et al. [44]. Hence, evaluating the associations of stress indices with GY under different environments is necessary. Therefore, the objectives of the current study were to (i) evaluate the performance of yield and yield attributes of Thai lowland rice genotypes under TWS and identify stress tolerant genotypes using stress indices; (ii) to identify promising stress indices which are correlated with GY under WW and TWS conditions for their use as rapid identifiers in rice crop breeding program for enhancing drought stress tolerance.

## 2. Results

### 2.1. Effect of Water Stress on Yield Performance and Productivity

**Figure 1:**
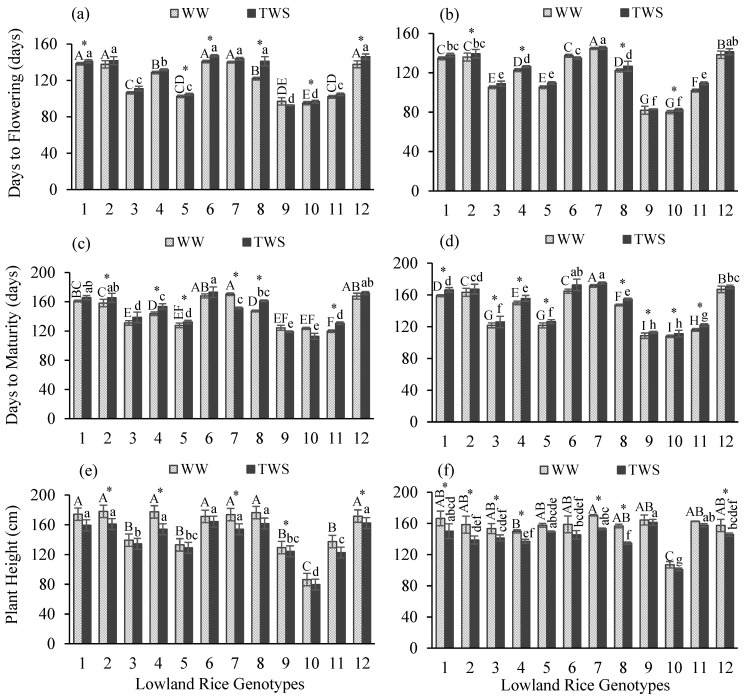
Days to flowering (**a**,**b**), days to maturity (**c**,**d**) and plant height (**e**,**f**) of twelve lowland rice genotypes under well-watered (WW) and terminal water stressed (TWS) conditions during 2018–2019 (**a**,**c**,**e**) and 2019–2020 (**b**,**d**,**f**). Vertical bars show ± standard errors for means of three repetitions. Capital letters represent the significant (*p* < 0.05) differences among genotypes in WW condition. Small letters represent the significant (*p* < 0.05) differences among genotypes in TWS condition. Centered stars above each pair of the bars represent the significance of parameters for each genotype under WW and TWS conditions.

**Figure 2:**
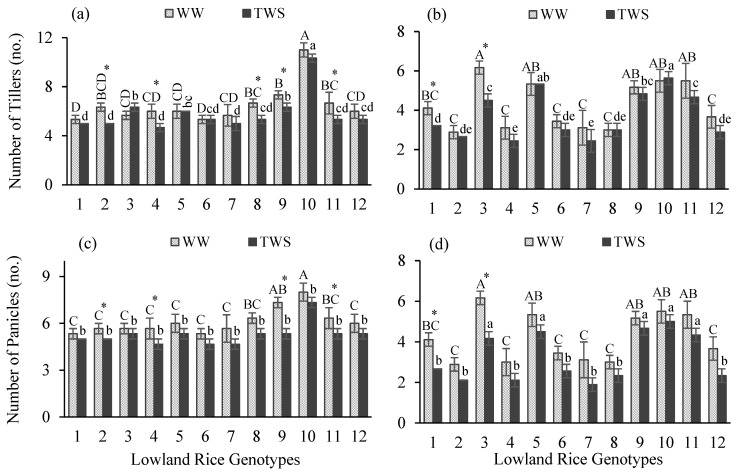
Number of tillers (**a**,**b**), and number of panicles (**c**,**d**) of twelve lowland rice genotypes under well-watered (WW) and terminal water stressed (TWS) conditions during 2018–2019 (**a**,**c**) and 2019–2020 (**b**,**d**). Vertical bars show ± standard errors for means of three repetitions. Capital letters represent the significant (*p* < 0.05) differences among genotypes in WW condition. Small letters represent the significant (*p* < 0.05) differences among genotypes in TWS condition. Centered stars above each pair of the bars represent the significance of parameters for each genotype under WW and TWS conditions.

**Figure 3:**
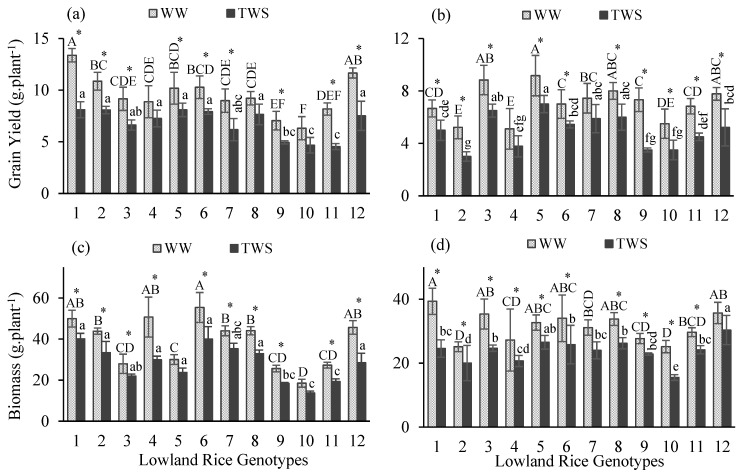
Grain yield (**a**,**b**) and biomass (**c**,**d**) of twelve lowland rice genotypes under well-watered (WW) and terminal water stressed (TWS) conditions during 2018–2019 (**a**,**c**) and 2019–2020 (**b**,**d**). Vertical bars show ± standard errors for means of three repetitions. Capital letters represent the significant (*p* < 0.05) differences among genotypes in WW condition. Small letters represent the significant (*p* < 0.05) differences among genotypes in TWS condition. Centered stars above each pair of the bars represent the significance of parameters for each genotype under WW and TWS conditions.

In this study, different lowland rice genotypes were assessed based on the performance of yield and yield attributes in response to terminal water stress (TWS) applied at the terminal crop growth stage. In both years, treatment and genotype effect resulted as highly significant different (*p* < 0.001) for most of the yield attributes except a non-significant difference for days to maturity (DM) under treatment effect in 2018–2019 (Table 1). Interactions of genotype and treatment effects indicated non-significant differences in both years, except for a significant difference for days to flowering (DF) (*p* < 0.05) and a highly significant difference for DM (*p* < 0.001) in 2018–2019 (Table 1). DF, number of tillers (NT), number of panicles (NP), grain yield (GY) and biomass were highly significant different; DM was moderately significantly different, whereas no significant difference was observed for plant height (PH) under the effect of years. Mean comparisons indicated that all tested genotypes differed and a significant variability in performance prevailed under well-watered (WW) and TWS conditions. TWS resulted in a delay in flowering duration (Figure 1a,b) of all genotypes except genotype 9 in the first year (Figure 1a). Flowering occurred 4 days earlier in genotype 9 (Table 2). Delay in flowering duration ranged 2–19 days in the first year while 1–4 days in the second year (Table 2). The maximum delay in flowering was observed for the top three genotypes 7, 12 and 6 by 19, 8 and 6 days in the first year and for 11, 8, 3, 4 and 5 by 7 and 4 days in the second year, respectively. TWS caused delays in the maturity duration (Figure 1c,d) of most of the genotypes except for genotypes 7, 9 and 10 in the first year (Figure 1a). Genotypes 7, 9 and 10 matured earlier in the first year by 19, 5 and 11 days (Table 2). In the second year, maturity duration was increased for all genotypes under TWS (Figure 1d). The delay in maturity duration ranged 4–14 days in the first year while 3–8 days in the second year (Table 2). PH was reduced under TWS for all genotypes in both years (Figure 1e,f). PH was reduced 4–13% in the first year and 2–14% in the second year (Table 2). Reduction in PH was higher than 10% for genotypes 1, 2, 4, 7, 8 and 11 (Table 2). NT (Figure 2a,b) and NP (Figure 2c,d) were reduced under TWS (Figure 2). However, reduction in NT and NP ranged one-two tillers and panicles per plant (Table 2). No change was observed in NT of genotypes 1, 5 and 6 in the first year and genotypes 2, 5, 6, 8, 9 and 10 in the second year (Table 2). Genotypes 1 and 3 maintained their NP under TWS in the first year, whereas the NP of all genotypes were affected in the second year (Table 2). TWS caused decline in GY (Figure 3a,b) and biomass (Figure 3c,d) of all genotypes in both years (Figure 3). GY was decreased 17–45% in the first year, whereas 21–52% in the second year (Table 2). The GY of genotypes 1, 7, 9, 11 and 12 in the first year and GY of genotypes 2, 9, 11 and 12 in the second year decreased more than 30%, indicating a major decline in GY under TWS (Table 2). Similarly, biomass was reduced 20–41% in the first year and 15–38% in the second year (Table 2). Biomass reduction of genotypes 4 and 12 in the first year and genotypes 1, 3 and 10 in the second year was more than 30%, indicating a major decline in biomass under TWS (Table 2).

### 2.2. Association among Yield and Yield Attributes under Terminal Water Stress

**Figure 4:**
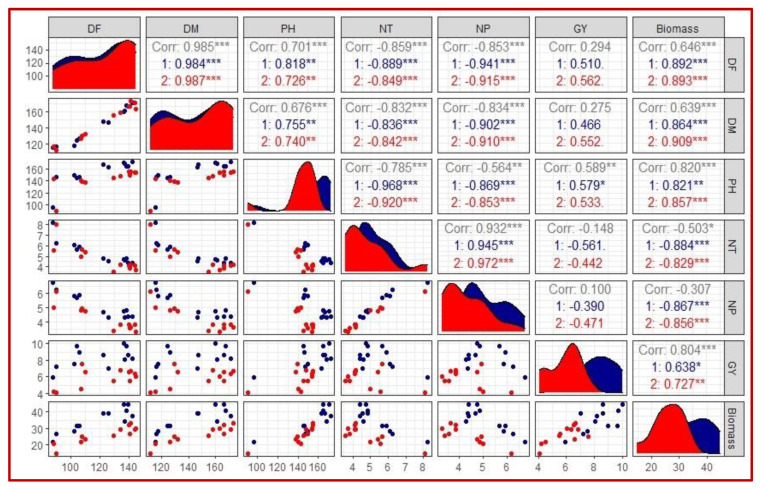
Combined correlation matrix, scatter plot and data distribution for yield and yield attributes of twelve lowland rice genotypes under well-watered (WW) and terminal water stressed (TWS) conditions. Diagonals indicate the distribution of each parameter. Scatter plots are shown in the bottom of diagonals. Values of correlations and significance are indicated with stars and are shown on the top of the diagonal. Values and stars in the blue color (1) indicate correlation among parameters in WW whereas, values and stars in the red color (2) indicate correlation among parameters in TWS conditions. DF: days to flowering, DM: days to maturity, PH: plant height, NT: number of tillers, PN: number of panicles, GY: grain yield, ***: highly significant (*p* < 0.001), **: moderately significant (*p* < 0.01), *: significant (*p* < 0.05).

Figure 4 indicates combined correlations among yield and yield attributes, including the DF, DM, PH, NT, NP, GY and biomass of twelve lowland rice genotypes. Under WW condition, highly positive associations among DF and biomass (0.89), DF and DM (0.98), DM and biomass (0.86), NT and NP (0.95), moderately positive associations among DF and PH (0.82), DM and PH (0.76), PH and biomass (0.82) and positive associations among PH and GY (0.56) and GY and biomass (0.64) were observed. Whereas highly negative associations among DF and NP (−0.94), DM and NP (−0.90), DM and NT (−0.84), PH and NP (−0.87), PH and NT (−0.97), NT and biomass (−0.88) and NP and biomass (−0.87) were detected. Under the TWS condition, highly positive associations among DF and biomass (0.89), DF and DM (0.99), DM and biomass (0.91), PH and biomass (0.86), NT and NP (0.97) and moderately positive associations among DF and PH (0.73), DM and PH (0.74) and GY and biomass (0.73) were observed. Whereas highly negative associations among DF and NP (−0.92), DF and NT (−0.85), DM and NP (−0.91), DM and NT (−0.84), PH and NP (−0.85), PH and NT (−0.92), NT and biomass (−0.83) and NP and biomass (−0.86) were detected (Figure 4).

### 2.3. Genotypic Classification Corresponding to Stress Indices

**Figure 5:**
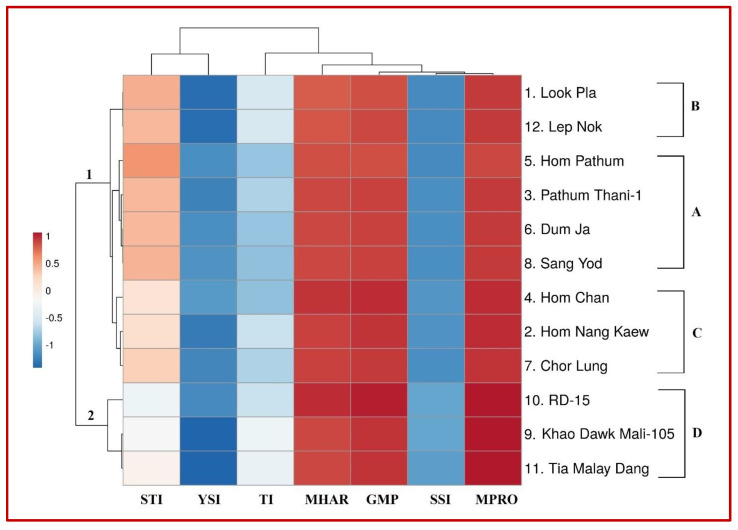
Heatmap of stress indices among twelve lowland rice genotypes under well-watered and terminal water stressed conditions. Group 1 refers to stress-tolerant genotypes, whereas group 2 refers to stress susceptible genotypes. Subgroup A is highly stress tolerant; subgroup B is stress tolerant; subgroup C is moderately stress tolerant, whereas subgroup group D is stress susceptible. Dark red and dark blue colors indicate higher correlation followed by light red and light blue with minimum or no correlation among genotypes and indices.

Seven stress tolerance indices, including SSI, GMP, STI, M_PRO_, M_HAR_, TI and YSI, were computed to distinguish stress-tolerant genotypes from stress-sensitive ones based on GY and RY and the promising values of stress indices under TWS conditions (Table 3). In addition, stress tolerance indices were also studied for hierarchical clustering using a heatmap (Figure 5) and the assessed genotypes were categorized into two main groups: (1) stress tolerant and (2) stress susceptible group and four subgroups (A–D). Subgroup A consisted of four genotypes with the highest GY, RY and stress indices values under TWS; hence, these genotypes could be considered as highly tolerant genotypes. Subgroup B consisted of two genotypes with higher GY, RY and higher stress indices values under TWS; hence, they could be considered as stress-tolerant genotypes. Subgroup C was moderate stress tolerant (three genotypes), as they exhibited intermediate values for GY, RY and stress indices. Subgroup D also consisted of three genotypes that exhibited lower values for GY, RY and stress indices; hence, these genotypes were considered stress susceptible genotypes correspondingly.

### 2.4. Association among Stress Tolerance Indices and Grain Yield

**Figure 6:**
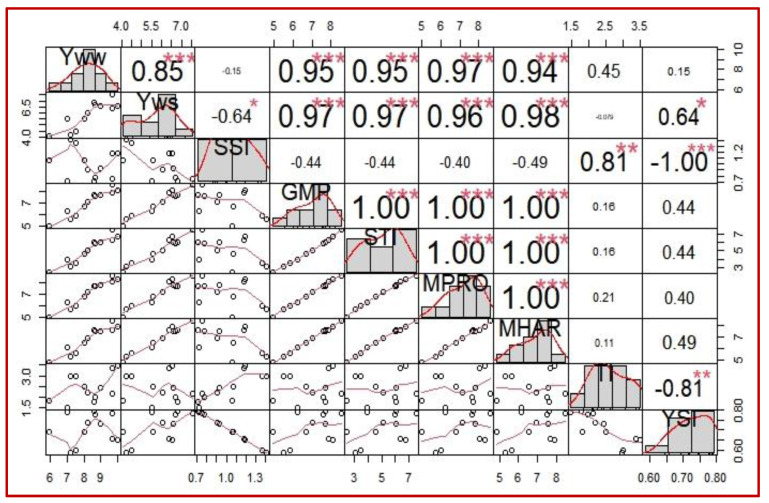
Correlation matrix (Pearson’s) of grain yield under well-watered (Y_WW_), grain yield under terminal water stress (Y_WS_), stress susceptibility index (SSI), geometric mean productivity (GMP), stress tolerance index (STI), mean productivity index (M_PRO_), harmonic mean index (M_HAR_), tolerance index (TI) and yield stability index (YSI) for lowland rice genotypes. Values were taken as average from two growing years 2018–2019 and 2019–2020. Diagonals indicate the distribution of each parameter. Scatter plots with lines are shown in the bottom of diagonals. Values of correlations and significance levels indicated with stars are shown on the top of diagonals. Correlation coefficients are proportional to intensity of color and size of correlation values. ***: highly significant (*p* < 0.001), **: moderately significant (*p* < 0.01), *: significant (*p* < 0.05).

Highly positive associations were observed among Y_WW_ and Y_TWS_ (0.85), Y_WW_ and GMP (0.95), Y_WW_ and STI (0.95), Y_WW_ and M_PRO_ (0.97), Y_WW_ and M_HAR_ (0.94), Y_TWS_ and GMP (0.97), Y_TWS_ and STI (0.97), Y_TWS_ and M_PRO_ (0.96) and Y_TWS_ and M_HAR_ (0.98). Whereas Y_TWS_ and YSI (0.64) were positively and Y_TWS_ and SSI (−0.64) were negatively correlated (Figure 6). Correlation assessment among stress indices revealed that there were highly positive associations among GMP, STI, M_PRO_ and M_HAR_ (1.00), whereas there was a moderate positive association among SSI and TI (0.81). In contrast, a highly negative association among SSI and YSI (−1.00) and moderate negative association among TI and YSI (−0.81) were observed (Figure 6).

## 3. Discussion

Water stress is critical to rice crop productivity, especially in rainfed lowland environments. Rainfed lowland rice is vulnerable as it is dependent upon natural precipitation. Variability in seasonal rainfalls and the occurrence of hot, dry spells have increased in rainfed areas. According to Campozano et al. [45] and Spinoni et al. [46], water stress occurrence is expected to be more common, severe and extended as a result of variations in rainfalls due to climate change. Water stress due to climate change would impact on rainfed rice crop productivity. Rice is extremely sensitive to water stress [2,14,15] and rice productivity is significantly affected under terminal water stress (TWS). Different rice genotypes exhibit differential response to TWS, producing a range of grain yield (GY). Hence, it becomes critical to evaluate the performance of yield attributes and yield productivity of rice genotypes under TWS and to identify stress-tolerant genotypes. This strategy will help to stabilize the rice productivity under TWS occurrence and provide sufficient information for genotypic stress tolerance. Furthermore, identification of promising stress tolerance indices under well-watered (WW) and TWS could be useful for their use in rapid selection process for water stress tolerance in the rice crop breeding program. 

Twelve lowland rice genotypes were evaluated under WW and TWS conditions in the current experimental study to examine their responses and identify stress-tolerant genotypes. It was observed that all genotypes indicated significant variations in their performance for yield and yield attributes under WW and TWS conditions. Generally, in our study, day to flowering (DF) and day to maturity (DM) were increased and DF and DM were significantly positive and strongly correlated. TWS caused delay in panicle emergence; hence, delaying the flowering time of most of genotypes. Delayed flowering in rice was also observed under water stress by Davatgar et al. [47], Saikumar et al. [48] and Hussain et al. [49]. Late flowering in rice under TWS is considered as a common impact of TWS [50,51]. Delayed panicle emergence and longer grain filling duration increased the time to maturity, thus increasing the total irrigation water input under TWS. All genotypes consumed more water input in delayed maturity under TWS after resuming irrigation. Plant height (PH) was decreased for all genotypes possibly due to limited water availability resulting in reduced cell elongation. Reduction in the PH of rice genotypes under water stress has been reported in numerous studies [47,48,49,52,53]. Significant positive correlation was observed among PH and GY and biomass while significant negative associations were indicated among PH and number of panicles (NP) and number of tillers (NT). NT and NP were reduced for all genotypes under TWS in both years. Increase in tiller mortality with increased duration of water stress has been reported by Zain et al. [54]. According to Davatgar et al. [47], water stress at terminal crop stages alters the source to sink association, which results in a reduced number of panicles. NT and NP were highly correlated, which indicated that more tillers produced more panicles. Stress induced at the terminal stage significantly reduced GY and biomass of all genotypes. TWS increases spikelet sterility and reduced grain weight resulting in declined final GY. Reduction in final GY under various water stress levels have been reported in several studies [19,48,55,56]. Biomass of all genotypes was reduced under TWS. However, genotypes with higher biomass produced higher GY. Strong positive association among GY and biomass was observed, and our results were in line with the findings of Torres and Henry [53], Torres et al. [56] and Kumar et al. [55]. High variability among genotypes for their performance of yield and yield attributes indicated that the genotypes could be used in the rice crop breeding program to exploit specific plant attributes such as early maturity, shorter plant height, higher tillering capacity and better GY under TWS for improvement in drought tolerance.

Explored genotypes exhibited highly significant variability in their GY productivity under WW and TWS conditions, which demonstrated that studied genotypes possessed significant genetic variability. Genotypes were differentiated based on GY productivity, relative yield (RY) and performance of computed stress indices which were further categorized into stress tolerant, and stress susceptible groups based on hierarchical clustering. Subgroup A was highly stress tolerant; subgroup B was stress tolerant; subgroup C was moderately stress tolerant, whereas subgroup group D was found stress susceptible. Highly stress-tolerant genotypes indicated the highest GY, RY and improved indices under TWS, whereas tolerant genotypes indicated higher GY, RY and better indices. However, stress-susceptible genotypes indicated lowered GY, RY and inadequate performance for stress indices. According to GY and performance of stress indices, hierarchical clustering helped to identify similarly acting genotypes under evaluation. Highly significant and positive correlation observed among GY under WW and GY under TWS exhibited that genotypes that performed better in WW conditions also produced well under TWS. Similar findings were also reported by Raman et al. [57]. Strongly significant and positive associations of stress indices, GMP, STI, M_PRO_, M_HAR_ with GY under WW and TWS were observed, which indicated that GMP, STI, M_PRO_ and M_HAR_ were better performer and promising indices to evaluate rice genotypes under WW and TWS conditions. Raman et al. [57] found that GMP and STI were suitable indices in identifying entries under non-stressed and extreme water stressed conditions. GMP has also been reported [31] as a better predictor for GY under water stress when stress was applied at the flowering stage. SSI, TI and YSI were not correlated with GY under WW. SSI was negatively correlated, YSI was significant and positively correlated, whereas TI was not correlated with GY under TWS. Weak associations of SSI, TI and YSI indicated that these indices were not adequate for evaluating lowland rice genotypes under TWS. Anwar et al. [29] also found that SSI, TI and YSI were not appropriate predictors of GY under WW and stressed conditions for evaluating wheat genotypes for drought stress tolerance. GMP, STI, M_PRO_ and M_HAR_ have been found to be suitable stress indices to evaluate genotypes under WW and stressed conditions for various crops including rice, wheat, maize and soyabean. Therefore, it was concluded that GMP, STI, M_PRO_ and M_HAR_ were appropriate indices for their use as rapid selection criteria for screening stress tolerant lowland rice genotypes grown under water stressed conditions, especially when stress is applied at reproductive or terminal crop stages.

## 4. Materials and Methods

### 4.1. Plant Material

Twelve commonly cultivated Thai lowland rice genotypes including Look Pla (1), Hom Nang Kaew (2), Pathum Thani-1 (3), Hom Chan (4), Hom Pathum (5), Dum Ja (6), Chor Lung (7), Sang Yod (8), Khao Dawk Mali-105 (9), RD-15 (10), Tia Malay Dang (11) and Lep Nok (12) were used for assessment in this study. Germplasm for genotypes 2, 4, 6, 7, 8 and 11 were collected from Phatthalung Rice Research Center, Phatthalung, Thailand (7°33′59.0″ N, 100°07′32.7″ E) (https://ptl-rrc.ricethailand.go.th/address.php (accessed on 21 September 2021)). Germplasm for genotypes 3, 9 and 10 was collected from commercial seed market. Whereas seeds for genotypes 1, 5 and 12 were collected from farmers in Songkhla province, Thailand.

### 4.2. Site Description and Crop Management

**Figure 7:**
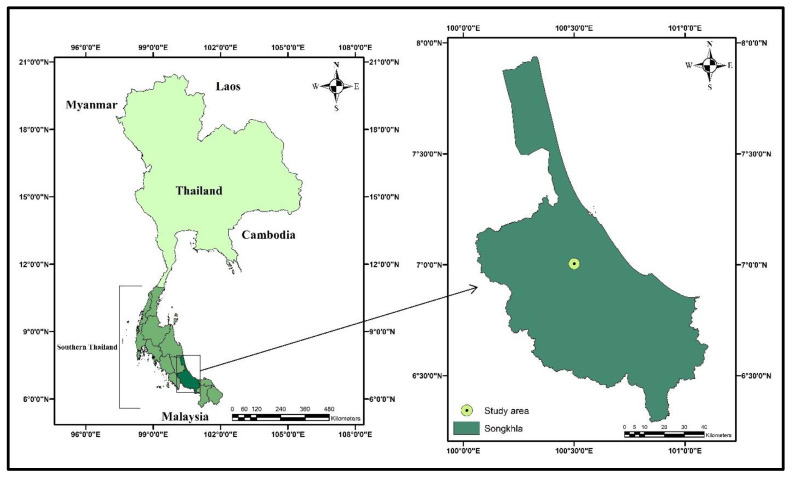
Experimental location at Faculty of Natural Resources, Prince of Songkla University, Songkhla, Thailand (Source: adapted from ArcGIS: *v*−10.5).

This research study was conducted in the sheds located at field research area (7°00′14.5″ N, 100°30′14.7″ E) of Faculty of Natural Resources, Prince of Songkla University, Hat Yai, Songkhla Province, in Southern Thailand (Figure 7) for two consecutive years during 2018–2019 and 2019–2020. Topsoil was prepared and a uniform soil sample was collected prior to soil filling in planting containers for soil properties analysis. Soil physicochemical properties observed for both years are indicated in Table S1. Planting was performed on 12 September 2018 and 2 September 2019 for 2018–2019 and 2019–2020, respectively. Completely randomized design (CRD) with three repeats was used to design the experiments for both years. Seeds were sown at 5 cm soil depth by direct seeding in containers having the capacity of 12 kg soil. Three plants were maintained in each container after thinning at seedling stage. Experiments were subjected to two treatments, including control under well-watered (WW) and drought under terminal water stressed (TWS) conditions. Each genotype in treatments was placed in separate group of containers. Automatic drip irrigation system, having the dripper head water flow capacity of 8 litters of water per hour, was installed to apply irrigation for specified time for each day. Plants in both treatments were irrigated equally till 75 days after planting (DAP). To induce TWS, irrigation was stopped at 75th DAP in TWS treatment only for 13 days until temporary wilting was observed, following which irrigation was resumed till maturity. Irrigation water amount as total water consumption for each genotype in each treatment for both growing years was calculated by dripper water flow capacity, irrigation time duration for each day and size of container used in experiments. Total water consumption for genotypes in WW and TWS conditions for each year is shown in Figure S1. Thinning, weeding, fertilization and insect pest management was completed through standard crop management practices.

### 4.3. Crop Data Collection

Days to flowering (DF) and days to maturity (DM) were recorded at 50% of panicle emergence and 50% plants at physiological maturity, respectively, from planting date. Plant height (PH) was measured from base of the stems to the flag leaf tip. GY and biomass were recorded by randomly selected three plants for each genotype from each replication as well as each treatment. Plants were hand-harvested, and number of tillers (NT) and number of panicles (NP) were counted per plant as an average from three plants. Grain and plant biomass samples were dried to obtain dry weight in an oven at 70 °C for different time durations till constant weight was observed.

### 4.4. Computation of Stress Tolerance Indices

Stress tolerance indices were computed to differentiate and identify stress tolerant genotypes from stress susceptible genotypes. GY under WW and TWS conditions was taken as average over 2 years of data to compute stress indices according to methodology adopted by Mansour et al. [6]. Seven different stress tolerance indices comprising stress susceptibility index (SSI) (1) [37], geometric mean productivity (GMP) (2) [32], stress tolerance index (STI) (3) [32], mean productivity index (M_PRO_) (4) [33], harmonic mean index (M_HAR_) (5) [34], tolerance index (TI) (6) [35] and yield stability index (YSI) (7) [36] were computed. Mean relative yield (RY) indicates the performance of specific genotype in relation to other examined genotypes under similar level of water stress. Hence, RY under TWS was calculated as GY of each genotype under TWS divided by highest GY achieved in all genotypes. Genotypes with higher GY under WW and TWS, higher RY and exhibiting promising values for stress tolerance indices were classified as stress tolerant genotypes.

(1) $$\mathrm{Stress}\;\mathrm{Suceptibility}\;\mathrm{Index}\;\left(\mathrm{SSI}\right)=\left(1-\frac{Y_{\mathrm{TWS}}}{Y_{\mathrm{WW}}}\right)/\;D\;$$

(2) $$\mathrm{Geometric}\;\mathrm{Mean}\;\mathrm{Productivity}\;(\mathrm{GMP})=\sqrt{Y_{\mathrm{WW}\;}\times Y_{\mathrm{TWS}}}$$

(3) $$\mathrm{Stress}\;\mathrm{Tolerance}\;\mathrm{Index}\;\left(\mathrm{STI}\right)=\left(Y_{\mathrm{TWS}\;}\times {\;Y}_{\mathrm{WW}}\right)\;/\;\mathrm{aww}$$

(4) $$\mathrm{Mean}\;\mathrm{Productivity}\;\mathrm{Index}\;\left(M_{\mathrm{PRO}}\right)=\left(Y_{\mathrm{TWS}}+Y_{\mathrm{WW}}\right)\;/\;2$$

(5) $$\mathrm{Harmonic}\;\mathrm{Mean}\;\mathrm{Index}\;\left(M_{\mathrm{HAR}}\right)=2\left(Y_{\mathrm{WW}}\times {\;Y}_{\mathrm{TWS}}\right)\;/\;\left(Y_{\mathrm{WW}}+Y_{\mathrm{TWS}}\right)$$

(6) $$\mathrm{Tolerance}\;\mathrm{Index}\;\left(\mathrm{TI}\right)=\left(Y_{\mathrm{WW}}-{\;Y}_{\mathrm{TWS}}\right)$$

(7) $$\mathrm{Yield}\;\mathrm{Stability}\;\mathrm{Index}\;\left(\mathrm{YSI}\right)={\;Y}_{\mathrm{TWS}}\;/{\;Y}_{\mathrm{WW}}$$

where, Y_TWS_ = mean yield under terminal water stressed (TWS) condition, Y_WW_ = mean yield under well-watered (WW) condition, D = environmental stress intensity, which is 1 (mean yield of all genotypes under TWS/mean yield of all genotypes under WW condition) and aww is an average value for all examined genotypes for grain yield under WW conditions.

### 4.5. Analysis of Data

Data collected from 2 years of experiments was used to test the significance of results and mean comparisons in R software. Two-way analysis of variance (ANOVA) was performed for yield and yield attributes of all genotypes from three replicates with effect to applied treatments. The effect of years among 2018–2019 and 2019–2020 was also examined. Mean comparisons were made by using the least significant difference (LSD) and *p*-value < 0.05 was considered as significantly different [58], which was represented using capital and small letters and stars. Pearson’s correlation analysis was used to correlate yield and yield attributes as well as computed stress tolerance indices. “Corr” and “GGally” packages of R program were used to compute correlation matrices and visuals. ClustVis [59] software was used to create heatmap and hierarchical clustering [58] for various stress indices taken as an average over two years.

## 5. Conclusions

Terminal water stress (TWS) significantly reduced the performance of yield and yield attributes. Studied genotypes were found unique in their yield potential as they reflected different responses under well-watered (WW) and TWS conditions. Genotypes Look Pla (1), Pathum Thani-1 (3) Hom Pathum (5), Dum Ja (6) Sang Yod (8), and Lep Nok (12) were found water stress tolerant as they produced relatively higher grain yield (GY), promising values for stress indices and improved performance under TWS. The performance of stress tolerant genotypes was less affected under TWS as compared to stress susceptible genotypes. Hence, these genotypes are potentially recommended for sustaining yield productivity in such environments where TWS occurrence is predicted, especially in southern Thailand. Stress-tolerant genotypes could be used in obtaining better GY under TWS and for improvement in drought tolerance. Strong associations of GMP, STI, M_PRO_ and M_HAR_ with GY under WW and, especially under TWS conditions, indicated that these indices could be used to indicate stress tolerance in rice crop breeding programs for a rapid selection process.

## Figures and Tables

**Table 1:** The analysis of variance for days to flowering (DF), days to maturity (DM), plant height (PH), number of tillers (NT), number of panicles (NP), grain yield (GY) and biomass (BM) of twelve lowland rice genotypes.

Year	Traits	Treatment (T) Effect	Genotype (G) Effect	Interaction (T × G)	Year Effect
2018–2019	DF	***	***	*	***
	DM	ns	***	***	**
	PH	***	***	ns	ns
	NT	***	***	ns	***
	NP	***	***	ns	***
	GY	***	***	ns	***
	BM	***	***	ns	***
2019–2020	DF	***	***	ns	
	DM	***	***	ns	
	PH	***	***	ns	
	NT	**	***	ns	
	NP	***	***	ns	
	GY	***	***	ns	
	BM	***	***	ns	

***: highly significant (*p* < 0.001), **: moderately significant (*p* < 0.01), *: significant (*p* < 0.05), ns: non-significant.

**Table 2:** Changes in performance of yield and yield attributes of twelve lowland rice genotypes under terminal water stressed conditions. Changes in days to flowering (DF) and days to maturity (DM) are presented by difference in days. Changes in number of tillers (NT) and number of panicles (NP) are presented by difference in numbers (no.), whereas changes in plant height (PH), grain yield (GY) and biomass (BM) are presented by % difference.

Genotypes	2018–2019							2019–2020						
	DF	DM	PH	NT	NP	GY	BM	DF	DM	PH	NT	NP	GY	BM
	Days	Days	%	no.	no.	%	%	Days	Days	%	no.	no.	%	%
1	3	5	−9	0	0	−39	−20	3	7	−10	−1	−1	−25	−38
2	4	7	−10	−1	−1	−26	−24	3	4	−12	0	−1	−43	−20
3	5	8	−4	1	−0	−28	−21	4	4	−8	−2	−2	−26	−30
4	3	10	−13	−1	−1	−18	−41	4	6	−8	−1	−1	−26	−24
5	2	5	−3	0	−1	−21	−21	4	5	−5	0	−1	−24	−19
6	6	5	−4	0	−1	−23	−28	−2	8	−8	0	−1	−22	−24
7	4	−19	−11	−1	−1	−31	−20	1	4	−10	−1	−1	−21	−23
8	19	14	−8	−1	−1	−17	−25	4	7	−14	0	−1	−25	−22
9	−4	−5	−4	−1	−2	−30	−28	1	4	−2	0	−1	−52	−17
10	2	−11	−8	−1	−1	−26	−26	2	3	−6	0	−1	−36	−38
11	3	11	−11	−1	−1	−45	−29	7	6	−3	−1	−1	−34	−19
12	8	4	−5	−1	−1	−36	−38	3	3	−7	−1	−1	−33	−15

**Table 3:** Values of seven stress tolerance indices for lowland rice genotypes based on grain yield observed under well-watered and terminal water stressed conditions. (Values taken as average from two growing years 2018–2019 and 2019–2020).

Lowland Rice Genotypes		Y_WW_	Y_TWS_	RY_TWS_	SSI	GMP	STI	M_PRO_	M_HAR_	TI	YSI
1	Look Pla	10.02	6.55	0.87	1.19	8.10	6.75	8.29	7.92	3.47	0.65
2	Hom Nang Kaew	8.04	5.54	0.73	1.07	6.67	4.58	6.79	6.56	2.50	0.69
3	Pathum Thani-1	9.00	6.56	0.87	0.93	7.68	6.07	7.78	7.59	2.43	0.73
4	Hom Chan	7.00	5.52	0.73	0.72	6.21	3.97	6.26	6.17	1.48	0.79
5	Hom Pathum	9.68	7.55	1.00	0.75	8.54	7.51	8.61	8.48	2.13	0.78
6	Dum Ja	8.64	6.68	0.89	0.78	7.60	5.94	7.66	7.54	1.96	0.77
7	Chor Lung	8.22	6.03	0.80	0.91	7.04	5.10	7.12	6.96	2.18	0.73
8	Sang Yod	8.61	6.83	0.90	0.71	7.66	6.04	7.72	7.61	1.78	0.79
9	Khao Dawk Mali-105	7.19	4.22	0.56	1.41	5.51	3.12	5.71	5.32	2.97	0.59
10	RD-15	5.91	4.08	0.54	1.06	4.91	2.48	5.00	4.83	1.82	0.69
11	Tia Malay Dang	7.51	4.52	0.60	1.36	5.82	3.49	6.01	5.64	2.99	0.60
12	Lep Nok	9.72	6.37	0.84	1.18	7.87	6.37	8.04	7.69	3.35	0.66

Y_WW_ is mean yield under well-watered conditions, Y_TWS_ is mean yield under terminal water stressed conditions, RY**_TWS_** is relative yield under water stressed conditions, SSI is stress susceptibility index, GMP is geometric mean productivity, STI is stress tolerance index, M_PRO_ is mean productivity index, M_HAR_ is harmonic mean index, TI is tolerance index and YSI is yield stability index.

## Data Availability

The data presented in this study are available in this article and supplementary materials.

## Supplementary Materials

The following are available online at https://www.mdpi.com/article/10.3390/plants10122565/s1, Table S1: Details of soil properties analyzed for experimental soil for 2018–2019 and 2019–2020. Figure S1: Total amount of irrigation water consumed by lowland rice genotypes under well-watered (WW) and terminal water stressed (TWS) conditions during 2018–2019 (a) and 2019–2020 (b).
