# Supplementary material for: Responses of Lowland Rice Genotypes under Terminal Water Stress and Identification of Drought Tolerance to Stabilize Rice Productivity in Southern Thailand
Source: Plants (Basel). 2021 Nov 24;10(12):2565. doi: 10.3390/plants10122565 (PMC8706981; doi:10.3390/plants10122565)
Supplement: Supplementary file 1 [file plants-10-02565-s001.zip › plants-1410390-supplementary.pdf]

Supplementary Table

Table S1. Details of soil properties analyzed for experimental soil for 2018–2019 and 2019–2020.

Growing years		Texture	pH	Organic matter	Total N	Available P	Available K
		class	-	g. kg^{-1}	g. kg^{-1}	mg. kg^{-1}	mg. kg^{-1}
1	2018-19	sandy clay loam	4.77	4.73	0.34	13.03	41.19
2	2019-20		5.29	4.60	0.30	35.58	58.67

Supplementary Figure

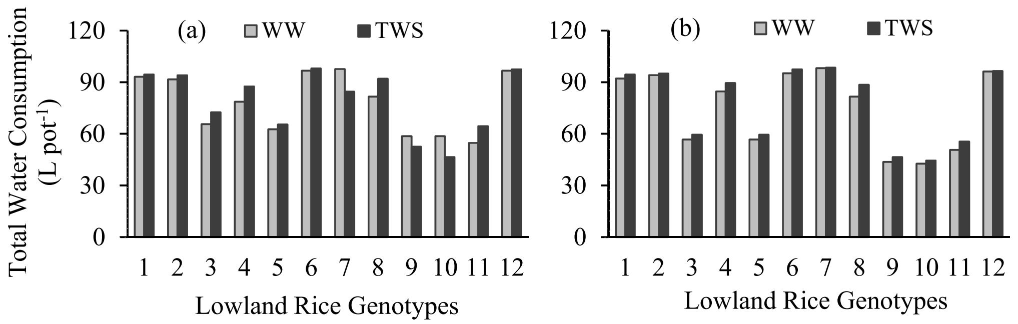

Figure S1. Total amount of irrigation water consumed by lowland rice genotypes under well-watered (WW) and terminal water-stressed (TWS) conditions during 2018–2019 (**a**) and 2019–2020 (**b**).
